# Interferon Inhibition Enhances the Pilot-Scale Production of Rabies Virus in Human Diploid MRC-5 Cells

**DOI:** 10.3390/v14010049

**Published:** 2021-12-29

**Authors:** Xiao Yang, Mingming Wan, Linjun Cai, Ali Hou, Bo Sun, Yan Zhou, Feng Gao, Weiheng Su, Chunlai Jiang

**Affiliations:** 1 National Engineering Laboratory for AIDS Vaccine, School of Life Sciences, Jilin University, Changchun 130012, China; yangxiao19900806@163.com (X.Y.); mingmingw77@163.com (M.W.); linjun_cai@jlu.edu.cn (L.C.); houal@jlu.edu.cn (A.H.); shrek1120@163.com (B.S.); julysec@jlu.edu.cn (Y.Z.); feng0215@gmail.com (F.G.); 2 Key Laboratory for Molecular Enzymology and Engineering of the Ministry of Education, School of Life Sciences, Jilin University, Changchun 130012, China

**Keywords:** inactivated vaccine, MRC-5 cell, IFN response, IFNAR1-deficient cell line, pilot scale production, rabies virus

## Abstract

Inactivated vaccines based on cell culture are very useful in the prevention and control of many diseases. The most popular strategy for the production of inactivated vaccines is based on monkey-derived Vero cells, which results in high productivity of the virus but has a certain carcinogenic risk due to non-human DNA contamination. Since human diploid cells, such as MRC-5 cells, can produce a safer vaccine, efforts to develop a strategy for inactivated vaccine production using these cells have been investigated using MRC-5 cells. However, most viruses do not replicate efficiently in MRC-5 cells. In this study, we found that rabies virus (RABV) infection activated a robust interferon (IFN)-β response in MRC-5 cells but almost none in Vero cells, suggesting that the IFN response could be a key limiting factor for virus production. Treatment of the MRC-5 cells with IFN inhibitors increased RABV titers by 10-fold. Additionally, the RABV titer yield was improved five-fold when using IFN receptor 1 (IFNAR1) antibodies. As such, we established a stable IFNAR1-deficient MRC-5 cell line (MRC-5^IFNAR1−^), which increased RABV production by 6.5-fold compared to normal MRC-5 cells. Furthermore, in a pilot-scale production in 1500 square centimeter spinner flasks, utilization of the MRC-5^IFNAR1−^ cell line or the addition of IFN inhibitors to MRC cells increased RABV production by 10-fold or four-fold, respectively. Thus, we successfully established a human diploid cell-based pilot scale virus production platform via inhibition of IFN response for rabies vaccines, which could also be used for other inactivated virus vaccine production.

## 1. Introduction

Since the 1960s, the technology of inactivated vaccine based on cell culture has developed rapidly. Because of its advantages including quick production process, fast development, low cost, high yields, and effective protection, it has gradually become the main form of vaccine against a variety of viruses [1], including varicella-zoster (VZV) [2], hepatitis A virus (HAV) [3], and rabies viruses (RABV) [4]. Recently, the COVID-19 inactivated vaccine based on cell culturing has been effective for the prevention and control of the epidemic situation in China [5].

Most inactivated vaccines are produced using Vero cells (African green monkey kidney cells), which have been used for more than 25 years for the production of vaccines against VZV, poliovirus, RABV, and most recently, COVID-19 [6,7,8,9]. Hundreds of millions of doses of Vero cell-derived vaccines are being distributed worldwide due to high viral replication and large-scale productivity [10]. Vero cells are an interferon (IFN)-deficient cell line, which could be used for achieving the high productivity of these viruses [11]. High yields of RABV production using Vero cells of 10^8^ fluorescence focus units (FFU)/mL under appropriate conditions have been achieved [12]. Due to these advantages, the inactivated vaccine based on Vero cells has become the mainstream product in the vaccine market.

Since Vero cells are monkey-derived, concerns exist over non-human DNA contamination during vaccine production. The DNA contamination exhibits a potential carcinogenic risk and increase the burden of vaccine purification and detection [13]. At present, one solution includes replacing Vero cells with human diploid cells [14]. Inactivated vaccines based on Vero cells, such as vaccines against VZV and HAV, are not approved in European countries and the US; however, they are used in developing countries [15]. In the field of rabies vaccines, purified Vero cell rabies vaccine (PVRV) has not been approved for the prevention of rabies in humans in the US. Although PVRV is approved in France, it is mainly sold in developing countries such as Asia. The human diploid cell rabies vaccine (HDCV) is marketed in France and the US.

In fact, the safety and effectiveness of human diploid cell inactivated vaccine have been fully demonstrated [16,17]. For example, the human embryonic lung fibroblast cells (MRC-5) line has become the source of many vaccines including VZV, HAV, and enterovirus 71 (EV71) inactivated vaccines because of its advantages of safety and production, especially with regard to the removal of the DNA contamination from host cells in vaccines [2,3,17]. Since the production of vaccines against most viruses including HAV and RABV using MRC-5 cells does not result in high yields, their development is slow. Thus, these cells are not widely used for inactivated vaccine production, thereby resulting in high prices. The yields of VZV and HAV-inactivated vaccine using human diploid cells were much lower than those using Vero cells [2,18]. At present, the output of HDCV in the Chinese market is 1/10th that of PVRV, and the price is 10 times higher than that of PVRV. Since it is a safer choice, the demand for HDCV has been increasing over the years. The 2020 Chinese HDCV production was expanded 10-fold as compared to that in 2010. However, the low yield has made it difficult to inoculate more people with HDCV. If the low production hurdle of human diploid cell is overcome, it will be of great significance for the promotion of inactivated virus vaccine production based on human diploid cells in developing countries.

Rabies is a fatal zoonotic disease that significantly impacts global public health, characterized by encephalopathy and generalized paresis [19]. Moreover, there are tens of thousands of human deaths annually, mainly in Asia and Africa [20]. Almost all cases of human rabies are caused by a bite from a rabid dog [21]. Lyssaviruses, such as RABV, are transported to the central nervous system (CNS) via retrograde axoplasmic flow and causes encephalitis [22], which has a 100% mortality rate if post-exposure prophylaxis is not administered in time. To combat these dire consequences, there has been tremendous progress in the control of rabies [23]. Safe and effective RABV vaccines for domestic animals as well as humans have been developed over the years [4]. Over the past 20 years, these vaccines have taken various experimental forms such as RABV glycoprotein subunit vaccines [24,25], DNA vaccines [26], and mRNA vaccines [27]. The most popular vaccines on the market at present are inactivated RABV vaccines.

The IFN response is one of the host’s primary defense mechanisms against viral infection. Viral infections trigger cellular type I IFN-α and -β (IFNα/β) responses. Secreted IFNα/β interacts with IFNAR1 and IFNAR2 receptors in an autocrine or paracrine manner, stimulating the JAK-STAT pathway to activate the expression of hundreds of IFN-stimulated genes (ISGs), many of which have antiviral activities [28]. Although the IFN system constitutes a powerful antiviral response, it rarely works to full capacity as viruses encode IFN antagonists [29]. Viruses which require cellular machinery for their replication have evolved different strategies to counteract the action of IFN, particularly by altering IFN-signaling and IFN-induced mediators. 

In this study, we aimed to identify the mechanism responsible for the low yield of RABV in MRC-5 cells and investigate strategies to overcome this problem in pilot-scale production.

## 2. Materials and Methods

### 2.1. Cell Culture, Virus, IFN Inhibitors, and Antibodies

MRC-5 cells were obtained from the American Type Culture Collection (Manassas, VA, USA; CCL-171) and cultured in Minimum Essential Medium (MEM; Invitrogen, Carlsbad, CA, USA) containing 10% fetal bovine serum, 100 IU/mL penicillin, and 100 mg/mL streptomycin at 37 °C in an atmosphere containing 5% CO_2_. Vero cells were obtained from the American Type Culture Collection and were cultured as monolayers in Dulbecco’s modified Eagle’s medium (DMEM) supplemented with 10% fetal calf serum, 100 IU/mL penicillin, and 100 mg/mL streptomycin at 37 °C in an atmosphere containing 5% CO_2_. The RABV strains used in this study were the PM1503 and aG strains provided by Chanchun BCHT Biotechnology Co. (Changchun, China). The virus titers were determined by 50% tissue culture infectious dose (TCID_50_) or plaque assays. Inhibitors of the IFN response (TPCA-1, Ruxolitinib) were prepared as 10 mM stocks in dimethyl sulfoxide (DMSO) and used at the indicated concentrations. Inhibitors were purchased from Selleck Chemicals. Antibodies against human IFN-α/β receptor 1 were gifted by the Key Laboratory of Infection and Immunity, Institute of Biophysics [30].

### 2.2. RABV Infection

MRC-5 or Vero cells were seeded into a 6-well plate. The number of cells before RABV infection were approximately 1.3 × 10^6^ per well. The inhibitors or antibodies were diluted with MEM medium. The MRC-5 cells were treated with inhibitors or antibodies, respectively, for 1 h at 37 °C before infection. MRC-5 cells were then infected with the PM1503 RABV strain, and Vero cells were infected with the aG RABV strain at different multiplicity of infection (MOI). Then, the cells were washed with PBS three times and cultured in MEM medium with 1% FBS after 4 h of infection. The cells and culture supernatant were collected at various time points after infection.

### 2.3. RNA Extraction and Quantitative RT-PCR

Total RNA was extracted using TRIzol reagent (Invitrogen, Carlsbad, CA, USA). One Step PrimeScript™ RT-PCR Kit II (Takara Bio, Inc., Kusatsu, Japan) was used with the CFX96 system (Bio-Rad Laboratories, Inc., Hercules, CA, USA) to quantify the expression of RABV, IFN-α, IFN-β, STAT1 (Signal transducing activator of transcription 1), and IRF7 (interferon regulatory factor 7) OAS1 (2′-5′-oligoadenylate synthase 1). For mRNA normalization, 18S rRNA was used. A total volume of 2 μL of sample RNA was added into 20 μL of reaction mix and subjected to the following conditions: 42 °C for 5 min, 95 °C for 10 s followed by 40 cycles at 95 °C for 5 s and 60 °C for 30 s. All reactions were performed in triplicate and analyzed using the 2^−^^△△Ct^ method. The sequences of the qRT-PCR primers are listed in Table 1.

### 2.4. Virus Production Assays

The content of surface viral glycoprotein was detected using the Diagnostic reagent for RABV glycoprotein antigen in the vaccine kit (Changchun Huili Biotech Co., Ltd., Changchun, China). The kit uses a double antibody sandwich enzyme-linked immunosorbent assay (ELISA). The 96-well plate was coated with anti-RABV monoclonal antibodies, and the content of virus glycoprotein was detected by anti-RABV glycoproteinase-labeled antibodies.

### 2.5. FFU Assays

In a 96-well plate, approximately 1 × 10^5^ dispersed Vero cells per well were seeded. The virus samples were subjected to a 5-fold serial dilution. Then, the samples (50 μL) of the serial dilution were added to a well in a 96-well plate. Virus and cell mixtures were incubated at 34 °C and 5% CO_2_ for 72 h. Cells were fixed with 80% acetone and treated with RABV-N-fluorescein isothiocyanate (FITC) antibodies at 37 °C for 1 h. The reaction was stopped with glycerol, and the RABV titer was calculated by the number of fluorescent cells (FFU/mL).

### 2.6. MRC-5^IFNAR1−^ Cell Line Construction by GenCRISPR™ System

The guidance RNAs (gRNAs) targeting the IFNAR1 region of the reference MRC-5 genome were designed using the Genetic Perturbation Platform (GPP) web tool (Copyright © 2020 Broad Institute). This report summarizes the development of a clustered regularly interspaced short palindromic repeat (CRISPR) knockout cell line by using GenCRISPR™ gene editing technology. Based on its genomic sequences on the database, a target gene sequence was analyzed, and target sites were located according to the manufacturer’s instructions for designing a targeting gRNA for the GenCRISPR™ system. Cleavage efficiency was evaluated by sequencing trace analysis using a CRISPR analysis tool (CAT). The gRNAs were cloned into plasmid PX458 (Addgene) behind the U6 promoter containing a Cas9 protein sequence. The PX458-gRNA plasmids or PX458 plasmids (NC) were transferred into MRC-5 cells at 680 V, respectively. MRC-5 Cells were maintained in MEM medium supplemented with 10% (*v/v*) FCS, 1% (*v/v*) penicillin–streptomycin, and 1% (*v/v*) l-glutamine. These cells were cultured in 96-well plates at a concentration of 0.8 per well and followed by puromycin (0.6 μg/mL) selection. Isogenic knockout cell clones were identified by Sanger sequencing (Table 2). MRC-5 cells, proven to produce mutations by sequencing results, were isolated and passaged to form three original MRC-5^IFNAR1−^ cell lines. After five passages, the endogenous mRNA from these cell lines was extracted, and the relative expression of the IFNAR1 gene was determined by qRT-PCR (Table 1).

### 2.7. Pilot Experiments

The MRC-5 cells, MRC-5^IFNAR1−^ cells, or Vero cells were seeded into 1500 square centimeter spinner flasks (Figure in Section 3.5). The number of cells before the viral infection was approximately 1 × 10^8^ per flask. The inhibitors were diluted with MEM medium. The MRC-5 cells were treated with 500 mL inhibitors for 1 h at 37 °C before infection. The MRC-5 or MRC-5^IFNAR1−^ cells were infected with the PM1503 RABV strain at an MOI of 0.05, and Vero cells were infected with the aG RABV strain at an MOI of 0.001. The cells were then washed with PBS three times and cultured in 3L MEM medium with 1% FBS after 4 h of infection. The cells and culture supernatant were collected at various time points after infection.

### 2.8. Statistical Analysis

All experiments were repeated in triplicate. The values were expressed as the mean ± standard deviation (SD). Statistical analysis was performed using a one-way analysis of variance (ANOVA) to compare the differences in t values between the experimental and control groups. Statistical significance was considered to be *p* < 0.05, and the *p*-values represented by asterisks was marked correspondingly in the figures, where * *p* < 0.05, ** *p* < 0.01, *** *p* < 0.001, **** *p* < 0.0001, and ns: *p* ≥ 0.05 non-significant.

## 3. Results

### 3.1. RABV Infection Activated IFN and IFN-Related Signaling Pathway in MRC-5 Cells

**Figure 1:**
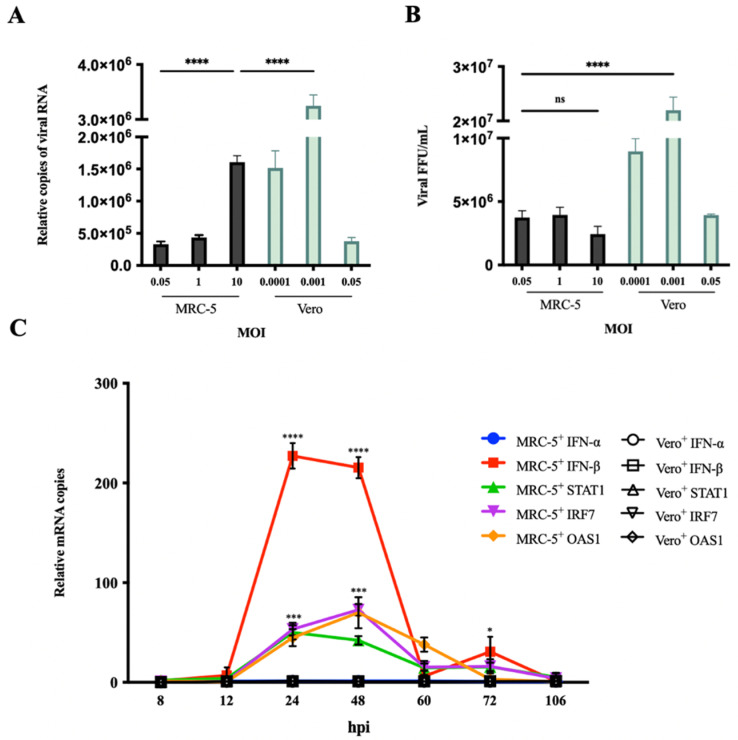
RABV infection activated IFN signaling pathway-related gene expression. (**A**,**B**) MRC-5 cells were infected with the PM RABV strain at MOI of 0.05, 1, and 10. Vero cells were infected with the aG RABV strain at an MOI of 0.0001, 0.001, and 0.05. RABV RNA expression and titer in culture supernatant were detected by qRT-PCR and FFU assays at 168 hpi. (**C**) MRC-5 cells were infected with the PM RABV strain at an MOI of 0.05. Vero cells were infected with the aG RABV strain at an MOI of 0.001. The total RNA from cells was extracted at 8, 12, 24, 48, 60, 72, and 106 hpi, and the mRNA expression of IFN-α, IFN-β, STAT1, IRF7, and OAS1 was detected by qRT-PCR. Data are shown as fold change in expression of mRNA for each IFN-α, IFN-β, STAT1, IRF7, and OAS1 compared with that of uninfected MRC-5 or Vero cells, respectively, at the indicated time point and normalized to the values for 18S rRNA. ^+^: RABV infection; * *p* < 0.05; *** *p* < 0.001; **** *p* < 0.0001; ns *p* ≥ 0.05 compared with Vero^+^ at the indicated time point.

To determine the difference in RABV production between MRC-5 cells and Vero cells, MRC-5 cells were infected with the PM RABV strains (adapted for replication in MRC-5 cells) at MOI values of 0.05, 0.1, 0.5, and Vero cells were infected with the aG RABV strains (adapted for replication in Vero cells) at MOI values of 0.0001, 0.001, and 0.05. The viral RNA and titer in the culture supernatant were tested by qRT-PCR and FFU assays at 168 h post infection (hpi) (Figure 1A,B). The results showed that MRC-5 cells had a significant reduction in RABV production compared with Vero cells. The viral titer in Vero cells reached its maximum at an MOI of 0.001 and was 10-fold higher than that of MRC-5 cells. Infection in MRC-5 cells at an MOI of 10 showed a significant increase in viral RNA expression but not a significant increase in viral titer. These results indicate that an enhancement of RABV production cannot be achieved by an increase in MOI. RABV MOI of 0.05 in MRC-5 cells and an MOI of 0.001 in Vero cells were determined to be the optimum MOI values for use in the following experiments. Additionally, the expression of IFN-α, IFN-β, and IFN signaling pathway endogenous genes, including STAT1, IRF7, and OAS1 in RABV-infected MRC-5 and Vero cells, was examined by qRT-PCR at 8 to 106 hpi, respectively (Figure 1C). The expression of these genes in MRC-5 cells was significantly upregulated compared with that in Vero cells at 24 and 48 hpi. Notably, IFN-β mRNA expression in MRC-5 cells showed a 200% increase compared to that in Vero cells. The IFN-α mRNA expression was not observed. This may be due to the fact that RABV, as a single-stranded RNA virus, enters cells and is mainly recognized by RIG-I, which can activate the expression of IFN-β [31,32]. These results suggest that the activation of the IFN signaling pathway genes acts as a crucial limiting factor to affect RABV production in MRC-5 cells. Inhibiting the activation of IFN response may be an effective approach to enhance RABV production in MRC-5 cells.

### 3.2. IFN Pathway Inhibitors Enhanced RABV Production in MRC-5 Cells

**Figure 2:**
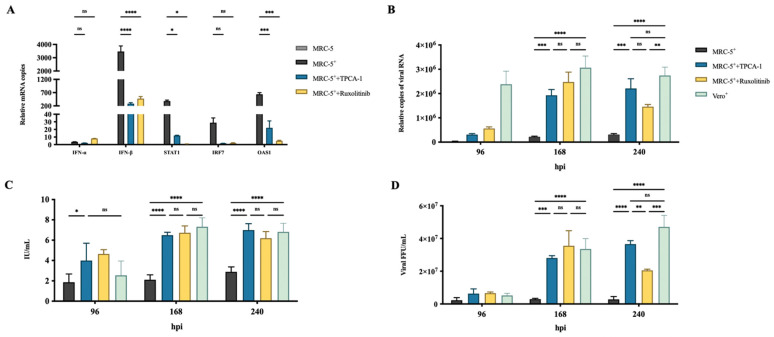
Treatment with IFN inhibitors enhanced RABV production in MRC-5 cells.MRC-5 cells were infected with the PM RABV strain at an MOI of 0.05. Vero cells were infected with the aG RABV strain at an MOI of 0.001. The MRC-5 cells were treated with 4 μM TPCA-1 or ruxolitinib 2 h before RABV infection. (**A**) Total RNA from cells was extracted at 24 hpi, and the expression of IFN-α, IFN-β, STAT1, IRF7, and OAS1 was detected by qRT-PCR. RABV mRNA expression, antigen G concentration, and viral titer in culture supernatant were measured by qRT-PCR (**B**), enzyme-linked immune-sorbent assay (ELISA) (**C**), and focus-forming unit (FFU) assays (**D**), respectively, at 96, 168, and 240 hpi. ^+^: RABV infection; * *p* < 0.05; ** *p* < 0.01, *** *p* < 0.001; **** *p* < 0.0001; ns *p* ≥ 0.05.

Two small molecules, nuclear factor kappa-B kinase-2 inhibitor, TPCA-1, acting on IFN-β promoter [33], and Janus kinase-1 inhibitor, ruxolitinib, acting on the signal transmission of interferon receptor [34], were used to inhibit IFN responses. These inhibitors were incubated with the cells 2 h before viral infection, and then endogenous IFN-α, IFN-β, STAT1, IRF7, and OAS1 mRNA expression were examined by qRT-PCR analysis at 24 hpi (Figure 2A). The MRC-5 cells treated with TPCA-1 or ruxolitinib showed a significant reduction in the mRNA expression of IFN-β, STAT1, IRF7 and OAS1 compared with the untreated cells (Figure 2A), suggesting the effectiveness of the inhibitor. Subsequently, the viral RNA, antigen G concentration, and RABV titer in the culture supernatant were measured by qRT-PCR, ELISA, and FFU assays at 96, 168, and 240 hpi [35], respectively (Figure 2B–D). The results showed a significant enhancement in viral RNA, G concentration, and titer at 168 and 240 hpi compared with the untreated cells, indicating an increase in RABV production. There was no significant difference in RABV production between MRC-5- and Vero-infected cells. These results confirm that IFN response is an important limiting factor of RABV production. Inhibiting the activation of IFN response can enhance RABV production in MRC-5 cells.

To explore the conditions of optimum IFN inhibitor usage for further improving RABV yields, yields at different inhibitor concentrations, MOI values, and virus harvest times were subjected to orthogonal testing (Table 3). We observed that virus production reached its maximum at an MOI of 0.1 and inhibitor concentrations of 1 μM at 240 hpi, and at an MOI of 0.05 and inhibitor concentrations of 4 μM at 168 hpi. These data provide potential guidance for applying IFN inhibitors for the production of RABV-inactivated vaccines.

### 3.3. Utilization of IFNAR1 Antibodies Enhanced RABV Production in MRC-5 Cells

**Figure 3:**
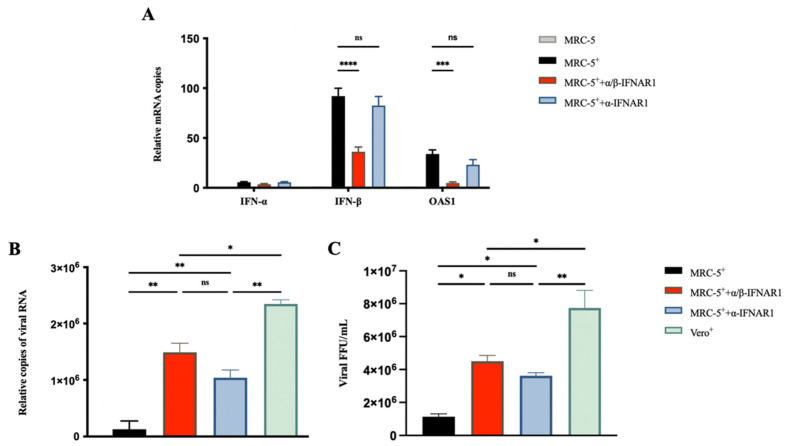
IFNAR1 antibodies enhanced RABV production in MRC-5 cells. MRC-5 cells were infected with the PM RABV strain at an MOI of 0.05. Vero cells were infected with the aG RABV strain at an MOI of 0.001. The IFNAR1 monoclonal antibodies α/β-IFNAR1 and α-IFNAR1 were incubated with MRC-5 cells 2 h before virus infection at a concentration of 1 ng/mL. (**A**) Total RNA from cells was extracted at 24 hpi, and the expression of IFN-α, IFN-β, and OAS1 mRNA was detected by qRT-PCR. Viral RNA (**B**) and titer (**C**) in the culture supernatant were detected by qRT-PCR and FFU assays at 168 hpi. ^+^: RABV infection; * *p* < 0.05; ** *p* < 0.01; *** *p* < 0.001; **** *p* < 0.0001; ns *p* ≥ 0.05.

The IFN-α/β receptor IFNAR1 mAbs α/β-IFNAR1 and α-IFNAR1 [30] were used to confirm the inhibition of RABV production by IFN in MRC-5 cells. The α/β-IFNAR1 mAb binds to both IFN-α and the IFN-β binding domain of IFNAR1, whereas α-IFNAR1 mAb only binds to the IFN-α binding domain. The antibodies were incubated with cells 2 h before viral infection, and then endogenous IFN-α, IFN-β, and OAS1 mRNA expression was examined by qRT-PCR at 24 hpi. The MRC-5 cells treated with α/β-IFNAR1 mAb showed a 60% reduction in the expression of IFN-β mRNA and an 80% reduction in the expression of OAS1 mRNA compared with the untreated cells (Figure 3A). On the contrary, the treatment of α-IFNAR1 mAb did not affect the expression of OAS1, as the virus infection did not activate the expression of IFN-α (Figure 3B). Additionally, the viral RNA and titer at 168 hpi in the culture supernatant were tested by qRT-PCR and FFU assays. The MRC-5 cells treated with α/β-IFNAR1 mAb showed a significant enhancement in RABV production (Figure 3B,C). α-IFNAR1 mAb treatment also showed an enhancement in RABV production. These results also indicated that the production of RABV is significantly enhanced in MRC-5 cells by the inhibition of IFN signaling pathway gene expression.

### 3.4. RABV Production Was Higher in MRC-5^IFNAR1−^ Cell Line

**Figure 4:**
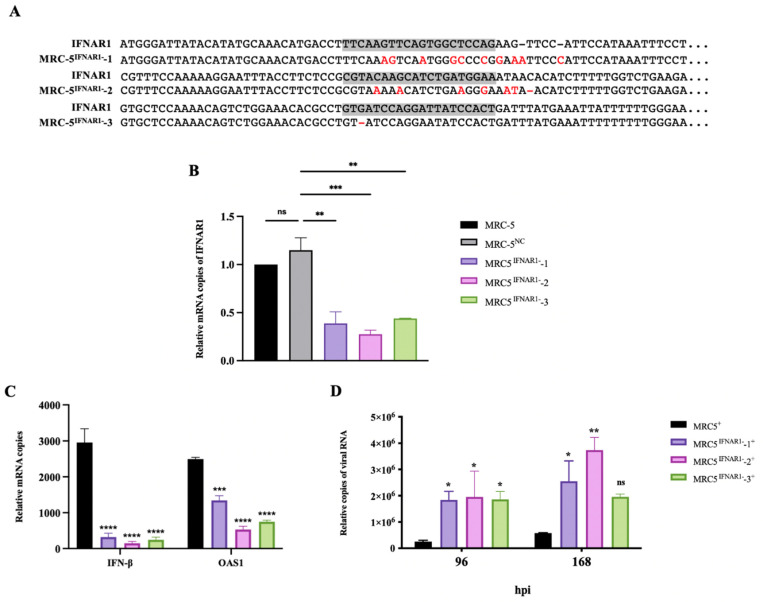
RABV production was enhanced in MRC-5^IFNAR1−^ cells. (**A**) Genomic DNA from three MRC-5^IFNAR1−^: gRNAs cell lines was extracted. The target sequence was amplified by PCR with IFNAR1 gRNA MRC-5^IFNAR1−^-1,2,3 primers in Table 4 and then was sequenced. The deficiency of IFNAR1 in three MRC-5^IFNAR1−^ cell lines were confirmed by sequencing trace analysis. The gRNA target area is marked in gray. Mutated bases are marked in red, and—means deletion. (**B**) Three MRC-5^IFNAR1−^ cell lines, MRC-5^NC^, and MRC-5 cells were cultured in 6-well plates to 80% confluence. Total RNA from cells was extracted, and the expression of IFNAR1 mRNA was detected by qRT-PCR. (**C**) Three MRC-5^IFNAR1−^ cell lines and MRC-5 cells were cultured in 6-well plates to 80% confluence. The cells were infected with the PM RABV strain at an MOI of 0.05. The total RNA from cells was extracted at 24 hpi, and the expression of IFN-β and OAS1 mRNA was detected by qRT-PCR. (**D**) Viral RNA in the culture supernatant were detected by qRT-PCR at 96 and 168 hpi. ^+^: Viral infection; ^NC^: non-targeting control; * *p* < 0.05; ** *p* < 0.01; *** *p* < 0.001; **** *p* < 0.0001; ns *p* ≥ 0.05 compared with MRC-5^+^ at the indicated gene or time.

To test the effects of genetic IFN signaling pathway defectiveness in MRC-5 cells affecting RABV yield, GenCRISPR™ gene editing technology was used to establish an IFNAR1-silent MRC-5 stable cell line (MRC-5^IFNAR1−^) and a non-targeting control MRC-5 stable cell line (MRC-5^NC^). Based on human IFNAR1 genomic sequences (GeneBank ID 3454), three targeting gRNAs were designed for GenCRISPR™ system (Table 4). By the transient transfection of GenScript CRISPR single-guide RNAs Cas9 in MRC-5 cells, the cleavage efficiency of three samples was 44%, 40%, and 48%, which was revealed by sequencing trace analysis using CAT CRISPR (Table 4). The mutations were identified by Sanger sequencing (Figure 4A). Three MRC-5^IFNAR1−^ cell lines, MRC-5^NC^, and MRC-5 cells RNA were extracted, and the expression of IFNAR1 mRNA was detected by qRT-PCR, respectively (Figure 4B). The IFNAR1 mRNA expression in each of the three MRC-5^IFNAR1−^ cell lines decreased by 62%, 73%, and 57% compared with that in MRC-5 cells. The decrease in mRNA expression level caused by mutation is likely due to NMD (nonsense-mediated mRNA decay) or NSD (no-stop decay) [36,37,38]. There was no significant difference between MRC-5^NC^ and MRC-5 cells. The MRC-5^IFNAR1−^-1, 2, 3, or MRC-5 cells were infected with RABV, and then endogenous IFN-β and OAS1 mRNA expression was examined by qRT-PCR at 24 hpi (Figure 4C). The IFN-β and OAS1 mRNA expression in each of three MRC-5^IFNAR1−^ cell lines decreased significantly compared with that in MRC-5 cells. The viral RNA in the culture supernatant of MRC-5^IFNAR1−^ or MRC-5 infected cells were also tested by qRT-PCR at 96 and 168 hpi. Viral production was significantly improved in each of three MRC-5^IFNAR1−^ cell lines compared with MRC-5 cells (Figure 4D). The MRC-5^IFNAR1−^-2 was the most productive cell line with a 6.5-fold increase in yields. These data suggest that the IFNAR1-defective cell lines can be used for RABV-inactivated vaccine production.

### 3.5. Use of IFN Inhibitor or MRC-5^IFNAR1−^ Cell Lines Enhanced RABV Production in Pilot Scale-Up Experiments

**Figure 5:**
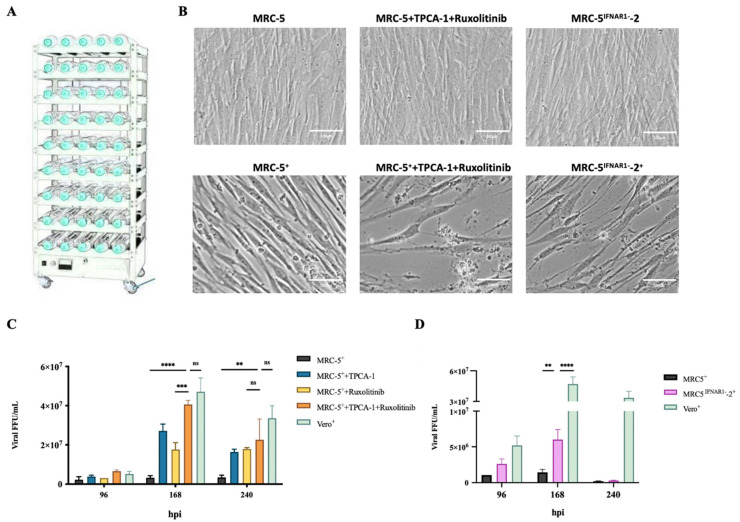
The IFN inhibitor or MRC-5^IFNAR1−^ cell lines enhanced rabies virus (RABV) production in pilot-scale experiments. (**A**) The pilot production platform consists of 30 spinner flasks with a surface area of 1500 cm^2^ and a constant temperature flask-rotating machine. The MRC-5^IFNAR1−^ cells and MRC-5 cells treated with both 2 μM TPCA-1 and Ruxolitinib 2 h before RABV infection were infected with the PM RABV strains at an MOI of 0.05 in 1500 square centimeter spinner flasks, respectively. Vero cells were infected with the aG RABV strains at a MOI of 0.001. (**B**) Representative cell status was recorded at 168 hpi. (**C**) The MRC-5 cells were treated with 4 μM TPCA-1, 4 μM Ruxolitinib, or both 2 μM TPCA-1 and Ruxolitinib 2 h before RABV infection. Viral titers in the culture supernatant were detected by focus-forming unit (FFU) assays at 96, 168, and 240 hpi. (**D**) The MRC-5 or MRC-5^IFNAR1−^ cells were infected with RABV. Viral titers in the culture supernatant were detected by FFU assays at 96, 168, and 240 hpi. ^+^: RABV infection; ** *p* < 0.01; *** *p* < 0.001; **** *p* < 0.0001; ns *p* ≥ 0.05.

We observed that three efficient approaches enhanced RABV production in MRC-5 cells. To further estimate their application in industrialization, IFN inhibitors and MRC-5^IFNAR1−^ cells were tested in pilot scale-up experiments for improving RABV production. The MRC-5 and MRC-5^IFNAR1−^ cells were passaged in 1500 square centimeter cell spinner flasks (Figure 5A). The MRC-5 cells were treated with 4 μM TPCA-1, 4 μM ruxolitinib, or both 2 μM TPCA-1 and Ruxolitinib 2 h before RABV infection. Subsequently, MRC-5 cells were infected with the PM RABV strain at a MOI of 0.05. The viral titer in the culture supernatant was determined by FFU assays at 96, 168, and 240 hpi. The viral production was increased by 8.3-, 5.2-, and 12.3-fold by TPCA-1, Ruxolitinib, or both TPCA-1 and Ruxolitinib, treatment, respectively, at 168 hpi (Figure 5C). The MRC-5^IFNAR1−^-2 cells were infected with the PM RABV strain at a MOI of 0.05. The viral titer in the culture supernatant was determined by FFU assays at 168 hpi. The viral production was increased 4-fold compared with that using MRC-5 cells (Figure 5D). Compared with IFN inhibitors, the MRC-5^IFNAR1−^ cell line did not perform efficiently and showed a substantial decrease at 240 hpi. Cell growth status of each experimental group was recorded at 168 hpi. Cell growth status showed that the IFN inhibitor and IFNAR1 silencing had no significant effect on the normal growth of MRC-5 cells. The difference in cytopathy shows the promoting effect of IFN inhibitor and IFNAR1 silencing on viral infection (Figure 5B). More than 80% cell death in the MRC-5^IFNAR1−^ cell group was detected at 240 hpi. This may be related to the higher population of MRC-5^IFNAR1−^ cells used. Compared with Vero cells, no significant difference in RABV production was observed in TPCA-1 or Ruxolitinib-treated MRC-5 cells. Addition of IFN inhibitors or the use of INFAR1-defective cell lines are potential solutions for the production of RABV vaccines in MRC-5 cells.

## 4. Discussion

Use of inactivated vaccines is an effective way to protect against a viral infections [1]. Inactivated viral vaccines produced using Vero cells are the most used product in the market. However, safety concerns posed by its residual non-human DNA need to be addressed. This has also led to such vaccine products being extremely demanding for DNA residual removal in quality control [39]. The demand for safer and more protective vaccines has given rise to a strategy to cultivate virus using human diploid cells such as MRC-5 cells and gradually replace Vero cells [40]. However, our results yielded a low RABV production in MRC-5 cells (Figure 1A). Thoulouze et al. [41] reported similar results: incubation with the challenge virus standard (CVS) RABV strain in a MRC-5 cell line at 24 hpi resulted in a low infectivity of approximately 30%, which resulted in low yields. Due to the low RABV production, human diploid cell-based inactivated RABV vaccines are expensive, limiting their mainstream production in the market. In 1977, Majer et al. [42] increased the infectivity of human diploid cells by screening for the appropriate viral strains. Nikolicetal et al. [43] reported that the inhibition of IFN-B gene expression increased RABV infection. However, neither of these studies improved the yield of RABV.

The cell’s innate immunity against viral infection involves the upregulated expression of antiviral proteins that are mediated by the type I IFN signaling pathway [44,45]. The IFN-induced antiviral response controls most viral infections and is indispensable for controlling viral infections in vertebrates [29]. The RABV P-protein targets both IFN induction and signaling [46]. The globular C-terminal domain of the P-protein (P-CTD) contains sites required for binding and activating STAT1, which, in turn, binds to downstream DNA, blocking the effects of IFN [47]. In 2002, Young et al. elucidated the practical applications of IFN-non-responsive cells in vaccine development and manufacture [48].

In this study, we found that RABV infection activated a strong IFN response in MRC-5 cells but almost none in Vero cells (Figure 1) [11]. This result is supported by several studies [49,50,51], suggesting that inhibition of the IFN response could result in better RABV production in MRC-5 cells.

We successfully enhanced RABV production by treatment with IFN inhibitors. The RABV production in MRC-5 cells treated with IFN inhibitors, TPCA-1 or Ruxolitinib, at 168 hpi increased yields by 10- and 13-fold, respectively, comparable to that in Vero cells (Figure 2). The receptor of IFN-α and IFN-β, IFNAR1 is the key node for IFN antiviral activity. In our study, the utilization of IFNAR1 mAbs, α/β-IFNAR1 mAb, and α-IFNAR1 mAb also significantly enhanced RABV production in MRC-5 cells (Figure 3). Additionally, we successfully established a stable MRC-5^IFNAR1−^ cell line via GenCRISPR™ gene editing technology. The IFN and OAS1 mRNA expression were inhibited, resulting in increased RABV production by 6-fold in the MRC-5^IFNAR1−^ cells (Figure 4). We showed that the MRC-5^IFNAR1−^ engineered cells could be used for inactivated RABV vaccine production. Additionally, the MRC-5^IFNAR1−^ cell line could be applied in the production of other viral vaccines as well as used as a detection cell line for viruses with low sensitivity to human diploid cells.

A marketed human diploid cell RABV vaccine produced by Chengdu Kanghua Biological Products Co. in China has a near 100% efficacy rate when used appropriately and greatly reduces the carcinogenic risk due to human residues [52]. However, due to its lower yield, this vaccine is significantly more expensive than Vero cell RABV vaccines. Nevertheless, in 2020, the use of this rabies vaccine expanded 10-fold compared to that in 2010. The development of human diploid cell inactivated RABV vaccines can be quickly promoted by improving viral yield in laboratory conditions or in the industrial production process of vaccines by using a convenient, inexpensive and suitable method for industrial production.

The successful use of inhibitors and MRC-5^IFNAR1−^ cell line in pilot scale-up experiments is significant in reducing the cost of production of inactivated virus vaccines. Inhibitors are inexpensive, less used, and calculated to contribute no less than $1 per vaccine to the rising cost caused by inhibitors. Residual inhibitor and clearance of inhibitors will be a hindrance to their application. We further constructed MRC-5^IFNAR1−^ cell line replace inhibitors. The MRC-5^IFNAR1−^ cell line will improve the yield of inactivated virus vaccines without changing vaccine manufacturing strategies. Although our data (Figure 5) show that MRC-5^IFNAR1−^ cell lines are not as effective as IFN inhibitors. This resulted from a 15 passage increase in cell passages caused by CRISPR and can be solved by reducing the original cell generation.

Research results are put into production in less than 1% of cases. Applying laboratory results to vaccine production is our ultimate purpose. There are currently many studies to improve the yield of virus in human diploid cells. Stewart et al. [53] reported that the IFN inhibitor, TPCA-1, can be used for respiratory syncytial virus (RSV) production in MRC-5 cells, where the productivity of RSV was significantly increased. Hamamoto et al. [54] reported increased yields of the influenza virus in MDCK cells by silencing IRF7 gene expression using siRNA. However, the protocols used in these studies were not used for industrial production. In the present study, the addition of an IFN inhibitor and the development of the MRC-5^IFNAR1−^ cell line was used for the production of RABV in a pilot study. The yield of RABV was significantly increased by the addition of IFN inhibitors to MRC cells or the use of MRC-5^IFNAR1−^ cell lines for culturing by 6- and 10-fold, respectively (Figure 5). Our results show the feasibility of these approaches in RABV production, laying the foundation for the potential mass production of human diploid cell inactivated RABV vaccines.

In summary, we demonstrated that it is feasible to increase the production of RABV vaccines in human diploid MRC-5 cells by blocking the IFN signaling pathway, either in the laboratory or in a pilot study. This study provides novel insights into the improvement of MRC-5 cell-based production strategies for RABV that could also be applied to other inactivated viral vaccines.

## Figures and Tables

**Table 1:** List of primers used for quantitative reverse transcriptase PCR.

Gene	Forward Primer (5′-3′)	Reverse Primer (5′-3′)
Rabies virus N protein	CAAGATGTGTGCYAAYTGGAG	AGCCCTGGTTCGAACATTCT
IFN-α	TTAGGATCCATGGCCTCGCCCTTT	CGCGAATTCGTTATTCCTTCCTCC
IFN-β	GTCTCCTCCAAATTGCTCTC	ACAGGAGCTTCTGACACTGA
STAT1	TTCTGTGTCTGAAGTGTAAGTGAA	TAACACGGGGATCTCAACAAGTTC
OAS1	AGAAGGCAGCTCACGAAACC	CCACCACCCAAGTTTCCTGTA
IRF7	GAGCCCTTACCTCCCCTGTTAT	CCACTGCAGCCCCTCATAG
18S	CTTAGAGGGACAAGTGGCG	ACGCTGAGCCAGTCAGTGTA
IFNAR1	GTAGAGGGGCGGTGAGAGCTA	CGCCATCGCCCCGTCCTAAG

**Table 2:** List of primers used for Sanger sequencing.

Gene	Forward Primer (5′-3′)	Reverse Primer (5′-3′)
IFNAR1 gRNA T1	TGCGAGCCTTTATCTTCTTGCC	CTGGCAGAACTGGGGTTAGA
IFNAR1 gRNA T2	TCTGTAACCTTAGCCCC	GACTGTTTTGGAGCACC
IFNAR1 gRNA T3	AACTACCCAGTGTGTCTT	CTCAAACCCTTAGGCTCA

**Table 3:** Supernatant rabies virus (RABV) antigen G concentration (IU/mL) at various time points post infection in MRC-5 with TPCA-1 treatment.

	MOI	96 hpi			168 hpi			240 hpi		
IU/mL		0.05	0.1	0.5	0.05	0.1	0.5	0.05	0.1	0.5
MRC-5^+^		2.22 ± 0.30	2.23 ± 0.27	2.76 ± 0.11	2.76 ± 0.11	2.26 ± 0.26	2.32 ± 0.13	2.32 ± 0.10	2.61 ± 0.29	3.36 ± 0.14
MRC-5^+^ + TPCA-1 (0.5 μM)		2.58 ± 0.22	2.09 ± 0.21	2.67 ± 0.12	2.87 ± 0.18	3.93 ± 0.19	4.49 ± 0.28	4.97 ± 0.25	4.81 ± 0.29	3.26 ± 0.34
MRC-5^+^ + TPCA-1 (1 μM)		2.36 ± 0.10	3.07 ± 0.17	2.76 ± 0.04	4.74 ± 0.11	4.85 ± 0.23	4.44 ± 0.38	6.94 ± 0.19	7.19 ± 0.50	4.13 ± 0.36
MRC-5^+^ + TPCA-1 (4 μM)		5.09 ± 0.40	4.74 ± 0.33	3.59 ± 0.38	7.12 ± 0.42	6.96 ± 0.44	4.27 ± 0.24	6.64 ± 0.56	5.78 ± 0.44	4.11 ± 0.22

**Table 4:** Sequences of IFNAR1 guidance RNA (gRNA) and evaluation of gRNA cleavage efficiency.

gRNAs	Sequence	Cleavage Efficiency
MRC-5^IFNAR1−^-1	CTGGAGCCACTGAACTTGAA	44%
MRC-5^IFNAR1−^-2	TTCCATCAGATGCTTGTACG	40%
MRC-5^IFNAR1−^-3	AGTGGATAATCCTGGATCAC	48%
